# Effect of Intravenous High Dose Vitamin C on Postoperative Pain and Morphine Use after Laparoscopic Colectomy: A Randomized Controlled Trial

**DOI:** 10.1155/2016/9147279

**Published:** 2016-10-30

**Authors:** Younghoon Jeon, Jun Seok Park, Suyoung Moon, Jinseok Yeo

**Affiliations:** ^1^Department of Anesthesiology and Pain Medicine, School of Dentistry, Kyungpook National University, Daegu, Republic of Korea; ^2^Colorectal Cancer Center, Kyungpook National University Medical Center, School of Medicine, Kyungpook National University, Daegu, Republic of Korea; ^3^Department of Anesthesiology and Pain Medicine, Kyungpook National University Hospital, School of Medicine, Kyungpook National University, Daegu, Republic of Korea; ^4^Department of Anesthesiology and Pain Medicine, Kyungpook National University Medical Center, School of Medicine, Kyungpook National University, Daegu, Republic of Korea

## Abstract

*Background and Objective*. Vitamin C has antioxidant, neuroprotective, and neuromodulating effects. Recently, it showed antinociceptive effect as a result of the antioxidant properties. Therefore, we designed this study to assess the effect of intravenous vitamin C on opiate consumption and pain in patients undergoing laparoscopic colectomy.* Methods*. A total of 100 patients were enrolled and allocated to receive 50 mg/kg vitamin C or placebo by intravenous infusion immediately after induction of anesthesia. Morphine consumption and scores of pain were assessed at 2, 6, and 24 h after completion of surgery.* Results*. There were 97 patients included in the analysis. Patients who received vitamin C had higher plasma concentrations of vitamin C at the end of surgery, significantly lower morphine consumption at the 2 h after end of surgery, and significantly lower pain scores at rest during first 24 h postoperatively. There was no significant difference between groups in side effects, fatigue score, or pain score during cough.* Conclusion*. This study shows high dose vitamin C infusion decreased postoperative pain during the first 24 h and reduced morphine consumption in the early postoperative period. Additional research needed to examine whether higher doses of vitamin C and longer infusion times can amplify these effects.

## 1. Introduction

The importance of providing adequate pain control during recovery from surgery cannot be overemphasized [1]. Laparoscopic surgical approaches might reduce postoperative pain associated with colorectal surgery [2], but postoperative pain management remains a complex challenge. Inadequate postoperative analgesia can lead to adverse events such as pneumonia, myocardial infarction, insomnia, and depression, thereby increasing morbidity and mortality and diminishing quality of life and patient satisfaction [3]. Opioids, which are still the most widely used class of analgesics in postoperative pain management, can increase the risk of postoperative nausea and vomiting (PONV) and can delay postsurgical recovery of gastrointestinal mobility. Recently, nonopioid analgesic alternatives have been introduced with the aim of improving pain management and limiting opioid-related side effects [4]. Vitamin C (ascorbic acid) is a water-soluble vitamin that has antioxidant, neuroprotective, and neuromodulating effects [5, 6]. Recent studies have shown that vitamin C supplementation might be a useful adjunct to pain management without significant side effects [7–9]. Therefore, we designed a randomized placebo-controlled trial to examine the effect of high dose vitamin C (50 mg/kg) on postoperative opiate consumption and pain scores in colon cancer patients during the first 24 h after laparoscopic colectomy. Postoperative fatigue and side effects were analyzed as secondary outcomes.

## 2. Patients and Methods

### 2.1. Patients

This study received approval from the ethics committee of Kyungpook National University Medical Center (ref: KNUMC_13-1048) and was registered with CRiS (Clinical Research Information Service, http://cris.nih.go.kr/, ref: KCT0000984, 2013.10.18). This randomized, double-blind study was conducted between November 2013 and October 2014. We enrolled colon cancer patients aged 20 to 75 years who were scheduled for elective laparoscopic colectomy under general anesthesia. We excluded patients who had a history of allergy to systemic opioids, substance use disorder, coagulopathy, chronic opioid use, sleep apnea, and analgesic use within 24 h. A member of the research team contacted the patients, explained the study protocol, and obtained informed consent for participation in the study. The day before surgery, all patients were taught how to use the patient-controlled analgesia (PCA) system (AIM plus; Hospira, Lake Forest, IL, USA) and how to rate pain intensity on the numeric rating scale (NRS), with 0 indicating no pain and 10 representing the worst pain imaginable (Table 1).

### 2.2. Protocol

Patients were randomly allocated to two groups using a computer-generated randomization table. Group allocation was concealed in sealed opaque envelopes. Baseline fatigue was assessed by NRS before induction of anesthesia. General anesthesia was administered according to a standard protocol and monitored according to American Society of Anesthesiologists guidelines. Blood samples (5 mL) for measurement of plasma vitamin C levels were taken from all patients before induction of anesthesia and prior to discharge from the operating room. Propofol (1 to 2 mg/kg) was administered for induction of anesthesia and rocuronium (0.4 mg/kg) was administered to facilitate intubation. Immediately after induction of anesthesia, a nurse who played no other role in the study selected an envelope for each patient and prepared an injection according to the group allocation. Patients in the vitamin C group received vitamin C 50 mg/kg (ascorbic acid 10 g/20 mL, Daewon, Korea) mixed with normal saline for a total injection volume of 50 mL, and those in the placebo group received normal saline 50 mL. The syringes were covered with black plastic and the solution was infused over 30 min using an infusion pump. Anesthesia was maintained using 4 to 8% desflurane in a mixture of 50% oxygen in air and remifentanil infusion. Additional doses of rocuronium were administered to maintain adequate muscle relaxation, and all patients received intravenous lactated Ringer's solution at rate 5 to 10 mL/kg/h during surgery. All patients received ramosetron 0.3 mg intravenously to prevent postoperative nausea and vomiting (PONV). All surgeries were performed using a 4-port technique with a minilaparotomy incision below the umbilicus at the camera port [10]. After the completion of surgery, the neuromuscular blockade was reversed with sugammadex 2 mg/kg. PCA was initiated for all patients upon arrival in the postanesthesia care unit (PACU). The PCA consisted of bolus doses of 1 mg morphine with a lockout time of 5 min and no basal infusion. Patients were instructed to push the PCA button any time they experienced pain. Rescue analgesia (50 mg tramadol injection) was given when the pain intensity exceeded VAS 4 for ≥30 min.

A research assistant who was blinded to the study group assignments checked patients for pain, fatigue, PONV, morphine consumption, and rescue analgesic requirement every 10 min during their stay in the PACU and again at 2, 6, and 24 h after discharge from the PACU to the ward.

### 2.3. Sample Size Calculation

We estimated a sample size on the basis of a pilot study that predicted a 24 h morphine requirement of 39.3 ± 20.5 mg for patients undergoing laparoscopic colectomy. We believed that a 30% reduction in morphine consumption after administration of vitamin C would be clinically relevant, and we determined that a minimum sample size of 48 patients per group would be necessary for 80% power to detect a 30% difference between the treatment group and the control group. A total of 50 patients per group were required to account for dropouts.

### 2.4. Statistical Analysis

Statistical analysis was performed using SPSS software version 21.0 (IBM Corporation, Armonk, NY). Continuous data were reported as mean ± standard deviation and analyzed using Student's *t*-test for comparisons between groups. Categorical data were reported as numbers and percentages and were analyzed using Chi square test. The differences in fatigue scores were compared using analysis of covariance (ANCOVA), which included baseline fatigue score as a covariate to account for individual variations in the level of fatigue. All reported *P* values are two-sided and the level of significance was set at *P* < 0.05.

## 3. Results

**Figure 1:**
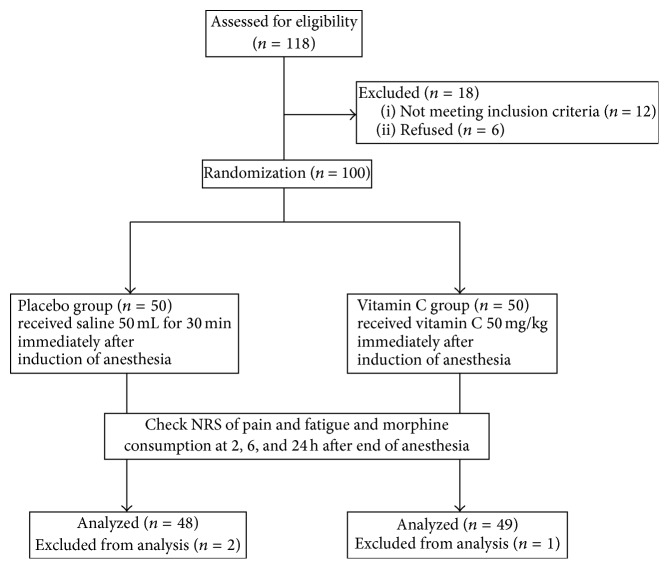
Flow chart describing recruitment, allocation, follow-up, and analysis. NRS, numeric rating scale.

We assessed 118 patients for eligibility. After excluding 18 patients, 12 who did not meet inclusion criteria (2 with allergy to morphine, 4 with a history of chronic opioid use, and 6 who were taking nonsteroidal anti-inflammatory drugs for osteoarthritis and spinal stenosis) and 6 who did not want to enter the study, 100 patients who were scheduled to undergo elective laparoscopic colectomy were included. Three of these were excluded from the final analysis due to complications (anastomotic leak and additional ileostomy). All operations were performed by a single surgeon (Figure 1).

**Figure 2:**
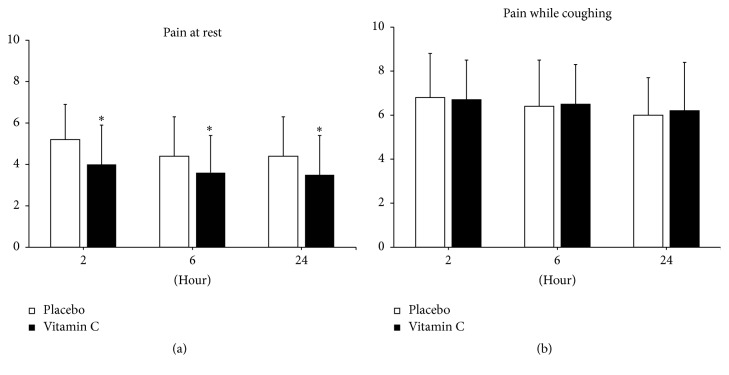
Pain scores at rest (a) and during coughing (b) by numeric rating scale at 2, 6, and 24 h after surgery (mean ± standard deviation). ^*∗*^ *P* < 0.05.

Data from a total of 97 patients were analyzed. There were no significant differences between groups in baseline patient characteristics, duration of surgery and anesthesia, intraoperative blood loss, and intraoperative fluid requirement. Postoperative pain scores at rest at 2, 6, and 24 h were significantly lower in the vitamin C group, but postoperative pain scores while coughing were not significantly different, and there was no difference between groups in postoperative fatigue scores. Morphine use during the first 2 h was significantly lower in the vitamin C group, but there was no significant difference between the two groups at 6 and 24 h. The baseline vitamin C concentration was slightly higher in the placebo group, but the difference was not significant, while the vitamin C concentration at the completion of surgery was significantly higher in the vitamin C group. Rescue analgesics were required more frequently in the placebo group, and the difference was significant compared to the treatment group (*P* = 0.00). The incidence of PONV and the length of hospital stay were similar between the two groups, and all patients were discharged home without significant complications within one to two days of resuming normal diets (Figure 2).

## 4. Discussion

**Figure 3:**
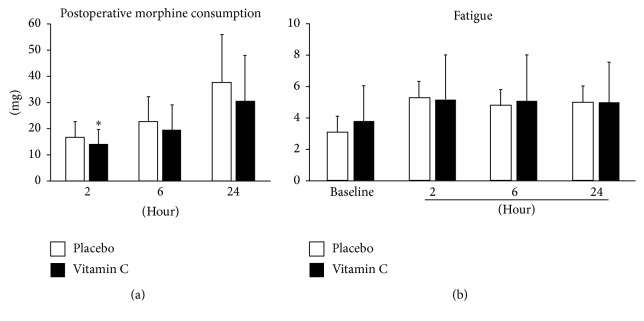
Postoperative morphine consumption (a) at 2, 6, and 24 h after surgery and numeric rating scores of fatigue (b) before surgery and at 2, 6, and 24 h after surgery. Data are shown as means ± standard deviation. ^*∗*^ *P* < 0.05.

Intravenous supplementation with 50 mg/kg vitamin C decreased postoperative pain at rest during the first 24 h and postoperative morphine consumption during the first 2 h after laparoscopic colectomy. There was no difference in cumulative morphine consumption between treatment and control groups at 6 h and 24 h. High dose vitamin C infusion also decreased the frequency of demand for rescue analgesics. The NRS pain scores during coughing and the NRS fatigue scores were comparable between the two groups at all time intervals (Figure 3).

Vitamin C is an essential micronutrient that serves as a cofactor in a number of enzymatic and chemical pathways [11]. The exact mechanism of the analgesic effect of vitamin C is not well understood, and several of the functions of vitamin C may contribute. As an antioxidant, vitamin C inhibits the formation of reactive oxygen and nitrogen species. Reactive oxygen species (ROS) are a known factor in neuropathic pain, and ROS scavengers can ameliorate mechanical discomfort [12]. A combination of antioxidants reduced levels of ROS and normalized tail flick latency in a mechanically induced animal pain model [13].

Vitamin C also has a neuromodulating function [14], specifically of dopamine- and glutamate-mediated neurotransmission [15]. Inhibition of the NMDA receptor by vitamin C decreases pain in chemically induced pain models [16]. Vitamin C is a key cofactor of dopamine b-monooxygenase and it is essential for norepinephrine biosynthesis. The conversion of dopamine to norepinephrine by dopamine b-monooxygenase is maximally efficient only in environments replete with extracellular ascorbic acid [17]. Finally, vitamin C is required for the release of noradrenaline and acetylcholine from synaptic vesicles [18]. These neurotransmitters are known to be the main components of the inhibitory pain pathway [19].

A plasma ascorbate concentration of <20 *μ*mol/L is accepted as a reliable indicator of an inadequate vitamin C reserve [11]. Vitamin C deficiency is commonly found in cancer patients [20], and patients who are scheduled for colon surgery generally need to be fasting for at least 24 h prior to surgery to ensure adequate bowel preparation; it is not surprising that the baseline plasma vitamin C concentration was <20 *μ*mol/L in both of our study groups. Laparoscopic colectomy generates oxidative stress and ROS-related injury has been attributed to abdominal inflation/deflation and use of carbon dioxide for the creation of pneumoperitoneum [21]. Response to surgical trauma, manipulation of the bowel, and exposure of the bowel to room air are also associated with ROS generation [22]. Increased levels of ROS and decreased pre- and postoperative plasma vitamin C concentrations can be offset by vitamin C supplementation, which replenishes antioxidants and increases plasma vitamin C levels [23] (Table 2).

The plasma vitamin C concentration increases rapidly after the start of an infusion and rapidly decreases once the infusion is finished. Peak plasma vitamin C concentrations occur immediately upon completion of a vitamin C infusion [24]. In our study, the vitamin C group had a higher plasma vitamin C concentration after surgery compared to the placebo group. The patients who received high dose (50 mg/kg) intravenous vitamin C at the start of surgery were apparently able to maintain higher plasma vitamin C concentrations throughout the surgery and in the early postoperative period, which may have been associated with decreased morphine consumption during the first 2 h after surgery and with the decreased pain scores at rest during first 24 h. While the single infusion of high dose vitamin C might have inhibited generation of ROS during surgery and contributed to reduced morphine consumption during the immediate postoperative period, the short half-life of the vitamin C does not support a sustained elevation of the plasma vitamin C concentration [24]. This could explain why there were no differences between groups in morphine consumption by 6 and 24 h postoperatively or in pain scores during cough at any time, and it suggests that a higher dose with a longer infusion time may be necessary to maintain adequate vitamin concentrations and sustained analgesic effects.

Suh et al. reported that intravenous infusion of vitamin C resulted in reduced fatigue among office workers and speculated that cancer patients and other groups who are at risk of vitamin C deficiency would exhibit even better responses [25]. However, our study did not support this. There was no significant reduction of fatigue score in the vitamin C group, despite higher plasma concentrations of vitamin C after surgery.

In conclusion, this study reports the effect of high dose intravenous supplementation of vitamin C on postoperative pain control. We found that infusion of vitamin C (50 mg/kg over 30 min) immediately after induction of anesthesia resulted in higher plasma concentrations of vitamin C at the completion of surgery and that it was associated with significantly reduced morphine consumption in the early postoperative period, along with significantly lower pain scores at rest during the first 24 h after surgery. Further research is warranted to examine whether higher doses and longer infusion times would amplify this effect.

## Figures and Tables

**Table 1:** Patient characteristics and perioperative data.

Group	Placebo	Vitamin C	*P* value
	(*n* = 48)	(*n* = 49)	
Age (y)	64.5 ± 10.6	65.4 ± 9.6	0.65
Sex (male/female)	29/21	28/22	0.84
Height (cm)	160.7 ± 8.8	160.2 ± 8.2	0.74
Weight (kg)	61.0 ± 9.4	61.8 ± 10.0	0.67
ASA PS (I/II)	16/34	15/35	0.83
Duration of surgery (min)	160.7 ± 46.0	160.2 ± 39.3	0.90
Duration of anesthesia (min)	186.5 ± 41.6	196.9 ± 39.0	0.20
Fluid (mL)	1324.0 ± 344.9	1363.3 ± 356.9	0.58
Estimated blood loss (mL)	212.8 ± 154.8	288.0 ± 223.9	0.62

Data are shown as mean ± standard deviation. ASA, American Society of Anesthesiologists Physical Status.

**Table 2:** Plasma vitamin C concentrations (*μ*mol/L) and frequency of postoperative rescue analgesics.

Group	Placebo (*n* = 48)	Vitamin C (*n* = 49)	*P* value
Vitamin C concentration			
Before surgery	15.0 ± 4.8	13.8 ± 4.6	0.55
End of surgery	12.1 ± 4.4	16.0 ± 3.7	0.03
Frequency of rescue analgesia	1.4 ± 1.0	0.8 ± 0.8	0.00

Data are shown as mean ± standard deviation.
